# Vapocoolant Analgesia for Breast Lymphoscintigraphy: A Prospective Clinical Trial

**DOI:** 10.2967/jnmt.123.266143

**Published:** 2024-06

**Authors:** Sanjit O. Tewari, Nina Tamirisa, Guofan Xu, Yang Lu

**Affiliations:** 1 Department of Nuclear Medicine, University of Texas M.D. Anderson Cancer Center, Houston, Texas; and; 2 Department of Breast Surgery, University of Texas M.D. Anderson Cancer Center, Houston, Texas

**Keywords:** breast, oncology, breast cancer, lymphoscintigraphy, vapocoolant

## Abstract

Breast lymphoscintigraphy is commonly performed before initial surgical intervention and surgical staging in the setting of breast cancer. Breast lymphoscintigraphy injections can often be quite painful and are routinely performed without any anesthesia or analgesia, thus representing a significant unmet need for the breast cancer population. Although vapocoolants have been previously available, they have typically been used on intact skin and not been recommended for sterile procedures. **Methods:** Thirty consecutive patients were enrolled in our prospective study of which 29 received vapocoolant analgesia in the setting of breast lymphoscintigraphy. Patients were given a postinjection questionnaire that included a self-reported pain score and boolean question regarding whether they would recommend vapocoolant for future patients. **Results:** The lymposcintigraphy procedure was successful in 100% of cases with an ipsilateral axillary node identified on average within 2.4 h of injection (median, 1 h; range 1–4.5 h). The average self-reported pain score was 1.98 (median, 1; range, 1–10). **Conclusion:** Vapocoolant analgesia in the setting of breast lymphoscintigraphy is feasible, does not appear to compromise lymphoscintigraphy, and appears to be associated with generally low self-reported pain scores.

## Visual Abstract

**Figure:**
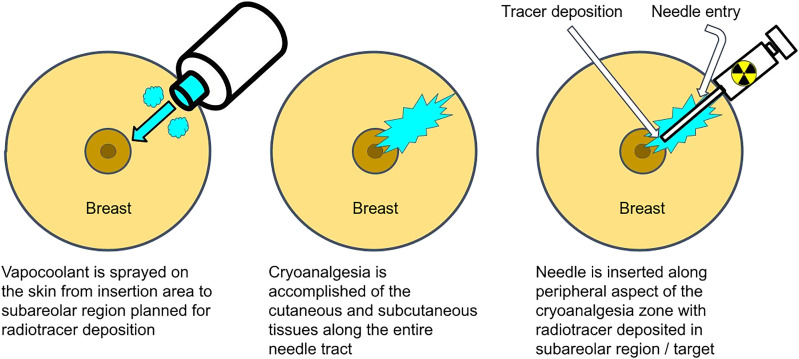

Breast lymphoscintigraphy is commonly performed before initial surgical interventions and surgical staging in the setting of breast cancer. Although breast lymphoscintigraphy may also be performed perioperatively under general anesthesia, by performing lymphoscintigraphy preoperatively, breast surgeons are often able to better plan their surgical staging procedures by prospectively being aware if there is indeed a draining lymph node of interest, the number of such lymph nodes, and the anatomic locations to investigate. In addition, performing breast lymphoscintigraphy as a separate preoperative procedure allows markedly increased time for radiotracer transit, as patients are often imaged for several hours and can be reassessed intraoperatively, with the surgery often scheduled for the following day.

Pain from breast lymphoscintigraphy is considered multifactorial, with the pathophysiology likely including stretching of skin layers and the acidity of the injectant, with the pH usually ranging from 3.5 to 6.0 (1). Several previously investigated interventions in breast lymphoscintigraphy include use of topical lidocaine (2) and buffered lidocaine (3*,*^4^), among others. However, use of intradermal buffered lidocaine, sometimes itself painful, requires 2 separate needle sticks and may require additional volume administration, which may accentuate skin stretching, whereas use of topical lidocaine requires significant time to be effective, which itself may create a myriad of workflow challenges. In addition, newer lymphoscintigraphy agents, specifically ^99m^Tc-tilmanocept, have shown no difference from ^99m^Tc-sulfur colloid in experienced pain (5), demonstrating the stubbornness of this unmet need among the breast cancer population.

Vapocoolants have been previously investigated in a myriad of applications, with the physiology of cryoanalgesia likely related to decreased speed of nerve conduction, decreased release of substances that produce local pain, possible interference of gate control mechanisms, decreased edema related to vasoconstriction, and even possible central nervous system effects related to endorphin release (1*,*^6^). Percutaneous probe-based cryoanalgesia has also been investigated in a myriad of situations, generally with regard to peripheral nerve pain (7*,*^8^). Although some studies previously have suggested that vapocoolants may not contaminate sterile fields (9*,*^10^), it was only recently that the Food and Drug Administration cleared a vapocoolant for sterile procedures, which instigated this clinical trial, the specific product being Nüm topical anesthetic spray, created by 623 Medical and distribution by Gilero.

## MATERIALS AND METHODS

Twenty-nine consecutive patients at a single subspecialty institution who had a diagnosis of breast cancer gave consent to be enrolled into our registered clinical trial and feasibility study (NCT05744557) and received vapocoolant analgesia as part of their breast lymphoscintigraphy examination. The local institutional review board approved the study, and all subjects gave written informed consent. Procedural details, lymphoscintigraphy results, and questionnaire results (self-reported pain on a scale of 1–10 and a yes/no question asking, “Would you recommend vapocoolant to other patients undergoing breast lymphoscintigraphy?”) were prospectively recorded and subsequently analyzed. The plan was to initially enroll 30 patients, but one patient was registered erroneously because of clerical error and, once recognized, was subsequently moved off trial.

The inclusion criteria included any patient referred for breast lymphoscintigraphy to be performed with the subareolar technique. Patients who requested a peritumoral injection were excluded.

For the subareolar injection, the intended needle tract was sterilized using alcohol swabbing. The radiotracer syringe was then removed from its lead casing just before vapocoolant administration to limit the time between vapocoolant administration and radiotracer injection and, therefore, rewarming. Vapocoolant was then administered to the skin at approximately 0.8–1.6 cm (2–4 in) from the intended needle tract, proceeding from the needle entry point to the area of subareolar radiotracer deposition in a slow undulating manner. If the skin frosted, vapocoolant administration was immediately ceased; otherwise, administration continued until the canister was empty (usually about 5 s).

Immediately on cessation of vapocoolant administration, the lymphoscintigraphy needle was inserted along the peripheral aspect of the vapocooled zone and then was advanced intradermally to the target subareolar region. Once in a satisfactory position, the radiotracer was administered expeditiously to limit the effects of rewarming and then was removed.

Planar anterior posterior and lateral images were obtained sequentially by the technologist until a sentinel lymph node was identified, with the first set of images usually acquired at 1 h after injection. Injections were performed on both same-day-surgery and next-day-surgery patients.

## RESULTS

All lymphoscintigraphy examinations were successful, with an ipsilateral lymph node identified in each patient (29/29) (Table 1). The average length of lymphoscintigraphy studies was 2.4 h (median, 1 h; range, 1–4.5 h). No complications were encountered (0/29). All patients recommended vapocoolant for breast scintigraphy (29/29), with an average self-reported pain score of 1.98 (median, 1; range, 1–10). In addition, 37.9% (11/29) experienced no pain at all, with a stated pain score of zero (Table 2).

**TABLE 1.** Results

Patient no.	Pain score (1–10)	Questionnaire (yes/no)	Side of breast	Lymphoscintigraphy result	Study length (h)	Complications
1	0	Yes	Right	Positive (axillary)	1.5	None
2	NA					
3	7	Yes	Left	Positive (axillary)	2	None
4	1	Yes	Right	Positive (axillary)	1.5	None
5	1	Yes	Left	Positive (axillary)	1.5	None
6	1	Yes	Left	Positive (axillary)	1.3	None
7	2	Yes	Right	Positive (axillary)	1.5	None
8	1	Yes	Left	Positive (axillary)	1	None
9	10	Yes	Left	Positive (axillary)	2.5	None
10	0	Yes	Left	Positive (axillary)	2	None
11	0	Yes	Right	Positive (axillary)	3.5	None
12	1	Yes	Left	Positive (axillary)	2	None
13	0	Yes	Left	Positive (axillary)	4	None
14	0	Yes	Right	Positive (axillary)	2.5	None
15	0	Yes	Right	Positive (axillary)	3	None
16	0	Yes	Right	Positive (axillary)	4	None
17	0	Yes	Left	Positive (axillary)	5	None
18	1	Yes	Left	Positive (axillary)	4.5	None
19	0	Yes	Right	Positive (axillary)	5	None
20	0	Yes	Left	Positive (axillary)	1	None
21	8	Yes	Right	Positive (axillary)	1	None
22	6	Yes	Left	Positive (axillary)	2.5	None
23	1.5	Yes	Right	Positive (axillary)	1.25	None
24	5	Yes	Right	Positive (axillary)	4	None
25	1	Yes	Left	Positive (axillary)	1	None
26	3	Yes	Left	Positive (axillary)	3.5	None
27	0	Yes	Right	Positive (axillary)	1.5	None
28	1	Yes	Right	Positive (axillary)	2.5	None
29	6	Yes	Left	Positive (axillary)	1.5	None
30	1	Yes	Left	Positive (axillary)	1.5	None

NA = not applicable.

**TABLE 2.** Result Analysis

Parameter	Average	Median
Pain (1–10)	1.98	1
Study length (h)	2.4	2

## DISCUSSION

The results of this clinical trial and feasibility study suggest that vapocoolant administration in the setting of breast lymphoscintigraphy is indeed feasible and, most importantly, does not affect the actual lymphoscintigraphy study, thus likely satisfying the first rule of clinical medicine: “Do no harm.” Furthermore, none of the patients seemed to have any strong aversion to vapocoolant administration, with all trial participants recommending vapocoolant administration for breast lymphoscintigraphy, which was a local concern before this feasibility study.

The effects of topical vapocoolants appear to involve predominantly the cutaneous and superficial subcutaneous tissues, making vapocoolants ideal for use in lymphoscintigraphy. Previous trials investigating vapocoolant use for puncture of an artery, generally considered a deeper structure, did not demonstrate any significant benefit (11).

Previous studies have characterized temperature thresholds with regard to cryoanalgesia, with the suggestion that analgesia be initiated at 13.6°C, with 2-point discrimination being compromised at 4°C and complete numbness occurring at 2°C. Such findings suggest that procedures should be performed expediently on vapocoolant administration and that the analgesic effect is likely quick to resolve (12*,*^13^). Such is why, as best as possible, lymphoscintigraphy was performed immediately after vapocoolant administration—to avoid confounding secondary to rewarming of the skin—and the lymphoscintigraphy dose was removed from its lead container just before vapocoolant administration to limit the intervening time. Moving forward, we plan to investigate several techniques that may optimize the effect of vapocoolant administration, including possible localization of the needle (specifically, piercing the skin and then placing the needle tip at the intended point of injection) before vapocoolant administration. Splitting the vapocoolant administration may also warrant exploration. We also plan to move forward with case-control trials in both breast cancer and malignant melanoma scintigraphy to better objectively elucidate the vapocoolant benefits.

Overall, our preliminary results suggest a rather low pain score of under 2 in a procedure that is otherwise considered exquisitely painful. We encourage other sites to conduct their own feasibility studies to help breast cancer patients experience a less painful treatment experience.

## CONCLUSION

Vapocoolant analgesia for breast scintigraphy is feasible, does not appear to compromise lymphoscintigraphy, is recommended by patients, and appears to be associated with overall low pain scores.

## DISCLOSURE

No potential conflict of interest relevant to this article was reported.

## Notes

KEY POINTS **QUESTION:** Is vapocoolant administration feasible in the setting of breast lymphoscintigraphy? **PERTINENT FINDINGS:** Vapocoolant administration in the setting of breast lymphoscintigraphy does not appear to be associated with any complications, does not appear to affect lymphoscintigraphy success, and appears to be associated with low patient-reported pain scores. **IMPLICATIONS FOR PATIENT CARE:** Vapocoolant administration may render breast lymphoscintigraphy more comfortable for patients.
